# Effects of sprint interval training on substrate oxidation in adults living with and without obesity: The i‐FLEX study

**DOI:** 10.14814/phy2.14916

**Published:** 2021-06-10

**Authors:** Benjamin H. Colpitts, Ken Seaman, Ashley L. Eadie, Keith R. Brunt, Danielle R. Bouchard, Martin Sénéchal

**Affiliations:** ^1^ Cardiometabolic Exercise & Lifestyle Laboratory Fredericton Canada; ^2^ Faculty of Kinesiology University of New Brunswick Fredericton Canada; ^3^ Department of Pharmacology Dalhousie Medicine New Brunswick Saint John Canada; ^4^ IMPART investigator team Canada

**Keywords:** adiposity, exercise training, fuel utilization, Type 2 diabetes mellitus

## Abstract

Metabolic flexibility is the ability to adapt substrate oxidation according to metabolic demand. Exercise increases fat oxidation responses in individuals living with obesity; however, limited research exists on the relationship between substrate oxidation and insulin sensitivity after sprint interval training (SIT). The primary objective was to investigate changes in substrate oxidation at rest and during submaximal exercise, and in insulin sensitivity after 4 weeks of SIT in individuals living with or without obesity. The secondary objective was to investigate correlations between changes in substrate oxidation and insulin sensitivity following SIT. Adults living with obesity (*n* = 16, body mass index (BMI) = 34.1 kg/m^2^ ± 3.8) and without obesity (*n* = 18, BMI = 22.9 kg/m^2^ ± 1.6) took part in a 4‐week SIT intervention. Participants completed three sessions of SIT per week, consisting of repeated sets of a 30‐s Wingate separated by 4 m of active recovery. Substrate oxidation at rest and during submaximal exercise was measured using VCO_2_/VO_2_. Insulin sensitivity was calculated using the Matsuda index. No difference in substrate oxidation at rest was observed for either group (*p* > 0.05), while a significant increase in fat oxidation was observed in individuals living with obesity [F(1,31) = 14.55, *p* = 0.001] during the submaximal exercise test. A significant decrease in insulin sensitivity was observed among individuals without obesity [F(1,31) = 5.010, *p* = 0.033]. No correlations were observed between changes in substrate oxidation and insulin sensitivity (*p* > 0.05). Following SIT, individuals living with obesity increased submaximal fat oxidation compared to individuals without obesity. No correlations were observed between substrate oxidation and insulin sensitivity. Thus, SIT impacts fat oxidation during exercise in individuals living with obesity while having no such influence on insulin sensitivity.

## Graphical abstract

The primary objective was to investigate changes in substrate oxidation at rest and during exercise, and changes in insulin sensitivity for individuals living with or without obesity following 4 weeks of SIT. Following SIT, individuals living with obesity increased fat oxidation during exercise compared to individuals without obesity. No correlation was observed between changes in substrate oxidation and insulin sensitivity.

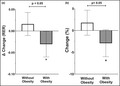

## 1 INTRODUCTION

According to estimates by the World Health Organization (WHO), the number of individuals living with obesity has nearly tripled worldwide over the last 40 years (Obesity & overweight,). This increased prevalence is concerning because of its association with Type 2 diabetes mellitus, cardiovascular disease, and musculoskeletal diseases (Obesity & overweight,). It is therefore important to identify effective training strategies to manage obesity and its related comorbidities.

Individuals living with obesity inefficiently alternate between fat and carbohydrate oxidation at rest and during submaximal exercise, which is often referred to as metabolic inflexibility (Battaglia et al., 2012; Kelley et al., 1999; Mittendorfer et al., 2009). In a study by Kelley et al. (1999) measuring fuel oxidation using blood gas measures across a catheterized leg, a significantly higher respiratory quotient (RQ) value was observed in fasted individuals living with obesity (0.90 ± 0.01) compared to individuals without obesity (0.83 ± 0.02; *p* < 0.01) (Kelley et al., 1999). Likewise, individuals living with obesity present inefficiencies at transitioning between fat and carbohydrate oxidation compared to individuals without obesity during acute bouts of submaximal exercise (Goodpaster et al., 2003; Pérez‐Martin et al., 2001). This is concerning as the literature suggests the combination of obesity and metabolic inflexibility (e.g., low fat oxidation at rest or during low‐intensity exercise) increase the likelihood of exhibiting a reduced insulin sensitivity; and thus, increasing the risk of developing Type 2 diabetes mellitus (Kelley et al., 1999; Kelley & Simoneau, 1994).

Increasing exercise levels is suggested to improve substrate oxidation in individuals living with obesity (Battaglia et al., 2012; Goodpaster et al., 2003; Jabbour & Iancu, 2017; Kanaley et al., 2001; Malin et al., 2013; Potteiger et al., 2008; Whyte et al., 2010). Continuous moderate‐to‐vigorous physical activity (MVPA) training has been thoroughly studied (Battaglia et al., 2012; Goodpaster et al., 2003; Kanaley et al., 2001; Malin et al., 2013; Potteiger et al., 2008), suggesting significant increases in fat oxidation at rest and during submaximal exercise. To date, few studies have investigated the impact of sprint interval training (SIT) on substrate oxidation in individuals living with obesity (Jabbour & Iancu, 2017; Whyte et al., 2010), which could be of interest due to the reduced time constraint (Korkiakangas et al., 2009). SIT entails exercising in a work:rest fashion where the working period is an all‐out (maximum) effort of exercise (Gibala & Hawley, 2017). Following only two weeks of SIT, a study revealed a significant increase in fat oxidation at rest (Whyte et al., 2010). In a randomized controlled trial, significant increases in fat oxidation at rest (11% improvement) and while cycling at 25 watts (26% improvement), 50 watts (21% improvement), or 75 watts (14% improvement) were reported following six weeks of SIT in individuals living with obesity (Jabbour & Iancu, 2017). These findings demonstrate the utility of SIT as a means of increasing fat oxidation at rest and during submaximal exercise. However, no study has investigated the difference in substrate oxidation between individuals living with and without obesity after short‐term SIT.

This knowledge gap is not trivial, since individuals living with obesity are at an increased likelihood of negative health outcomes including insulin resistance, cardiovascular disease, hypertension, and dyslipidemia compared to those without obesity (Nguyen et al., 2008; Riaz et al., 2018). As such, comparing body mass index (BMI) groups may yield important findings in relation to the impact of obesity status on cardiometabolic health outcomes. Many of the studies investigating changes in substrate oxidation are focused on continuous exercise (Battaglia et al., 2012; Goodpaster et al., 2003; Kanaley et al., 2001; Malin et al., 2013; Potteiger et al., 2008) and enhancing performance‐related outcomes (Goodpaster & Sparks, 2017). Thus, few studies have investigated SIT and substrate oxidation without limited generalizability (e.g., strictly males) (Whyte et al., 2010) and by using absolute workloads (e.g., 25, 50, and 75 watts) (Jabbour & Iancu, 2017). To our knowledge, no study has investigated the correlation between changes in substrate oxidation at rest and during submaximal exercise, and insulin sensitivity following SIT. Therefore, the primary objective was to investigate changes in insulin sensitivity and substrate oxidation at rest and during submaximal exercise in individuals living with or without obesity following a 4‐week SIT intervention. The secondary objective was to investigate whether changes in substrate oxidation following a 4‐week SIT intervention were correlated with changes in insulin sensitivity.

## 2 MATERIALS AND METHODS

### 2.1 Study design

**FIGURE 1:**
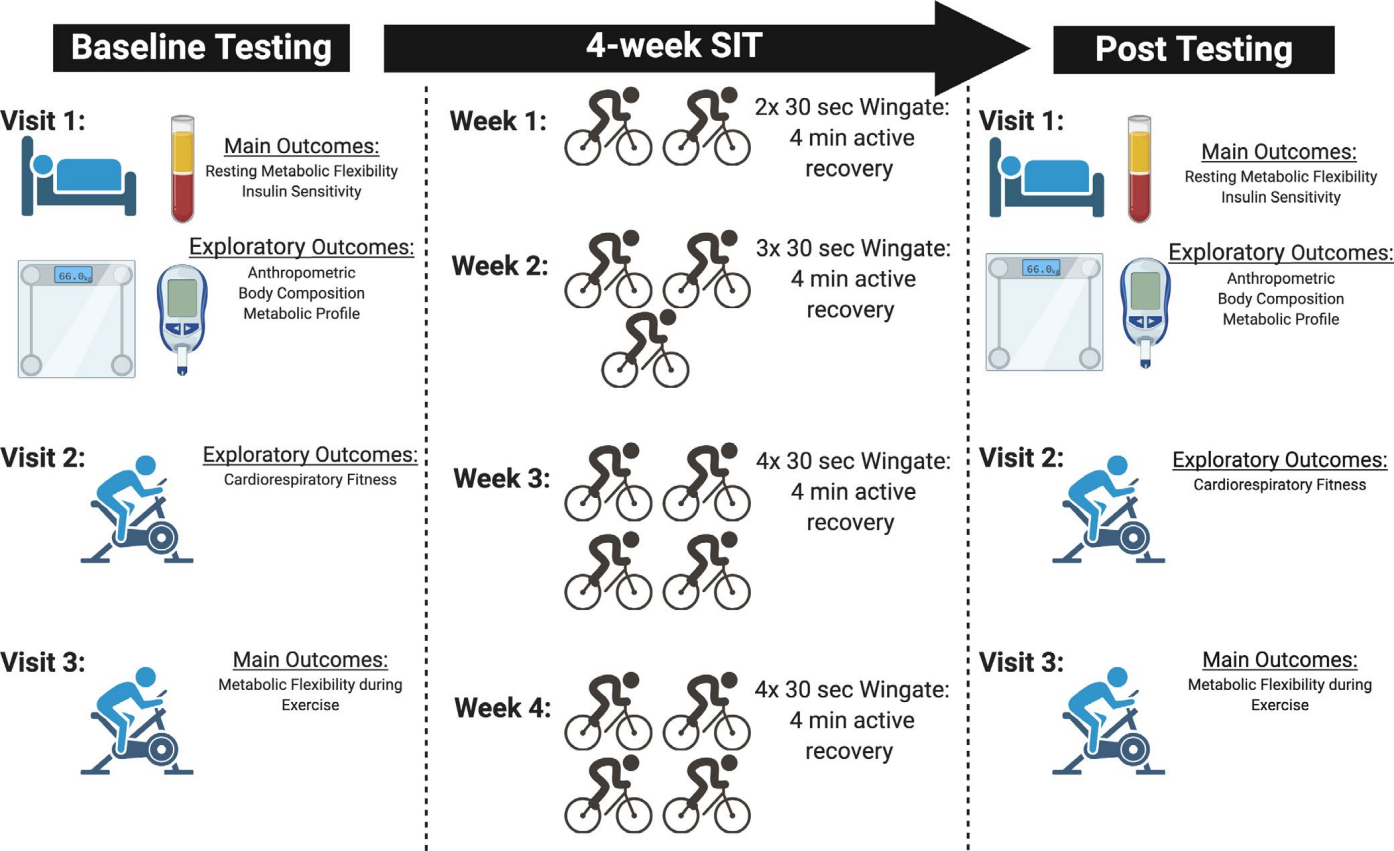
Experimental Timeline. (Created with BioRender.com)

Effects of sprint interval training on substrate oxidation in adults living with and without obesity – The i‐FLEX study was a single‐arm quasi‐experimental study comparing individuals living with obesity to individuals without obesity (Clinical Trial #: NCT03527446). A study overview is presented in Figure 1. Briefly, participants presented to the laboratory for baseline testing separated as three visits in a week's span. Participants then began four weeks of SIT within one week of the last baseline testing visit. Following the intervention, participants underwent follow‐up testing 72 h following their last intervention visit. This time frame was chosen as it has been suggested that oxygen consumption (Speakman & Selman, 2003) and insulin sensitivity (Roberts et al., 2013) are impacted for up to 72 h post‐exercise. All participants provided written and informed consent before participation. The project was reviewed and approved by the University of New Brunswick Research Ethics Board (REB 2018–058).

### 2.2 Inclusion criteria

Participants were eligible if they were between the ages of 19 and 60 years with a BMI greater or equal to 18.5 kg/m^2^ and below 25 kg/m^2^ (without obesity) or had a BMI greater or equal to 30 kg/m^2^ (living with obesity). The maximum age range was selected due to the challenge of recruiting participants above this age without chronic conditions while being simultaneously inactive and obese. To participate, individuals living with obesity had to be physically inactive which was based on not meeting the Canadian Physical Activity Guidelines from the Canadian Society for Exercise Physiology (CSEP): less than 150 min of MVPA per week in at least 10‐min bouts (CSEP, 2013). Physical activity levels were estimated using Piezo Rx Pedometers with pre‐determined thresholds (100 steps/min) for moderate intensity for one week (Marshall et al., 2013; Tudor‐Locke et al., 2005).

### 2.3 Exclusion criteria

Exclusion criteria included: (1) BMI and age outside of the predetermined thresholds, (2) individuals living with a condition that would impact their ability to perform SIT, (3) individuals living with diabetes or impaired glucose tolerance, (4) individuals that were taking medications that are known to impact insulin sensitivity and carbohydrate metabolism (i.e. corticosteroids or atypical antipsychotics), (5) individuals that experienced more than 10% weight loss or enrolled in a weight‐loss program over the past six months, and (6) individuals that take medication that are known to cause weight gain or weight loss.

### 2.4 Participant recruitment

**FIGURE 2:**
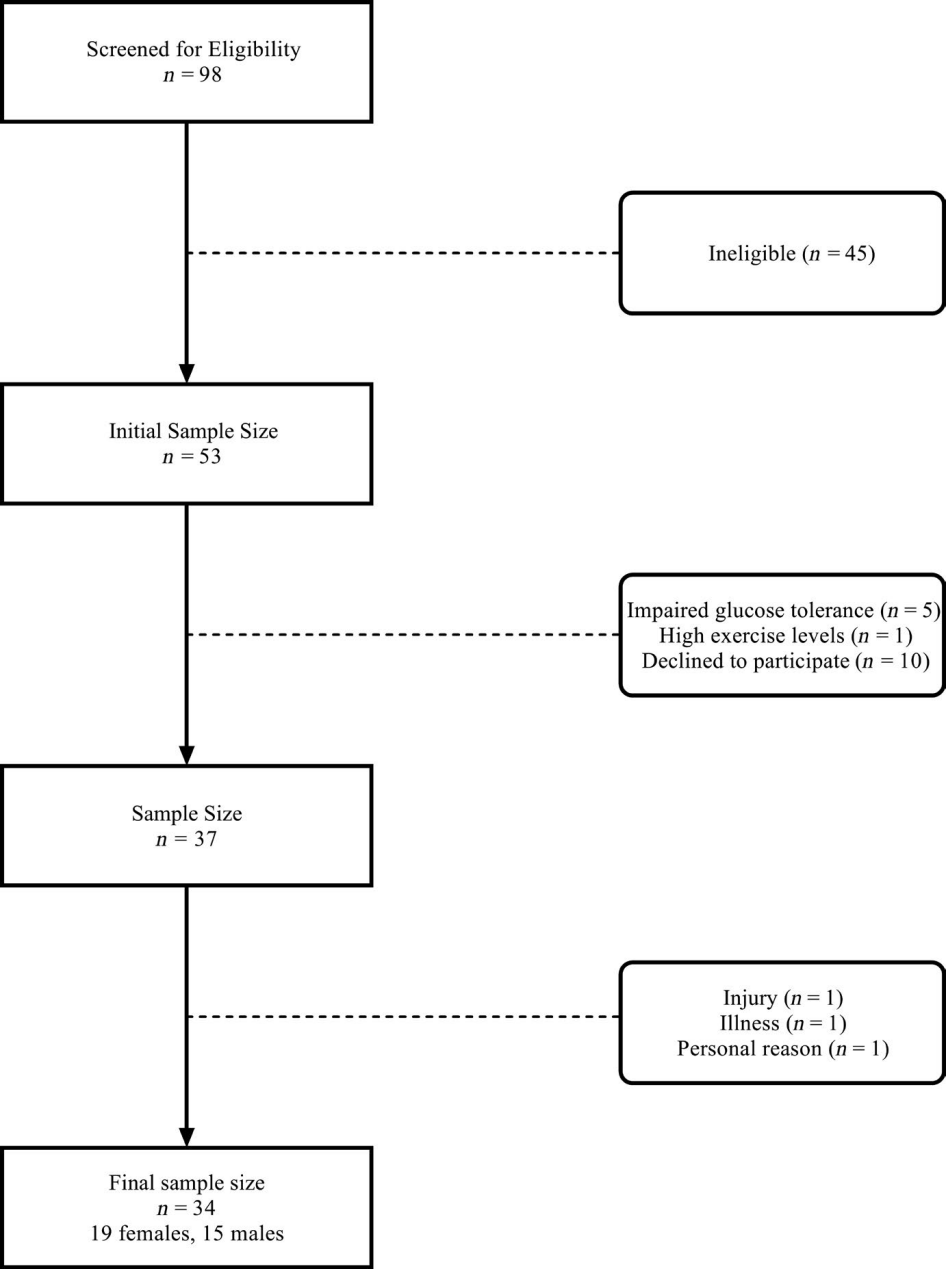
Participant Flowchart

Between June 2018 and December 2019, participant recruitment was performed using general community advertisements through social media platforms as well as news and notices platforms. A total of 98 participants were screened over the phone in which 45 were excluded because they did not meet the inclusion criteria. From this number, 53 participants came to the laboratory for baseline testing from which five were excluded because they demonstrated impaired glucose tolerance based on glucose values greater or equal to 7.8 mmol/L at the end (120 min) of an oral glucose tolerance test (OGTT) as recommended by previous literature (Nathan et al., 2007). An additional 10 participants declined to participate due to personal issues (e.g., scheduling concerns, travel, medical reasons, etc.) and one participant was excluded because they exceeded 150 min of MVPA per week. Therefore, a total of 37 participants were included in the study, from which three dropped out for the following reasons: injury or illness (unrelated to intervention), and personal reasons. A final sample size of 34 participants (19 females, 15 males) completed the study. An overview of participant recruitment can be seen in Figure 2.

### 2.5 Exposure variable—SIT intervention

Participants were required to perform four weeks of SIT on a Monark 874E Weight cycle ergometer. Participants attended supervised SIT sessions three times per week, with the number of intervals performed progressing from two to four over the duration of the intervention. All training sessions were supervised by the research staff. Briefly, participants started with 4 min of light‐intensity warm‐up prior to their first sprint interval. The work:rest ratio was 30 s:4 min; a 30‐s Wingate followed by 4 min of active recovery (at 59 watts; the minimum amount of wattage attainable on the cycle ergometer at 60 revolutions per minute). The drop load for the 30‐s Wingate was 7.5% of the participants total body weight to remain consistent with previous Wingate protocols (Bar‐Or, 1987; Driss & Vandewalle, 2013). The total exercise time per session ranged from 9 to 18 min as the intervention progressed. Research staff provided standardized encouragement throughout the 30 s Wingate. After exercise, a 5‐ min cool down was performed; then, seated heart rate and blood pressure were taken to ensure participants were properly recovered before exiting the laboratory. All 12 sessions were completed by the 34 participants.

### 2.6 Outcome measure—substrate oxidation

Substrate oxidation was estimated in two states: resting state and during an acute bout of moderate‐intensity exercise. Substrate oxidation at rest was estimated using the respiratory exchange ratio (RER) from a resting metabolic rate (RMR) test using the TrueMax 2400 Metabolic Measurement Cart (ParvoMedics). Participants entered the laboratory following a 12‐h fast and were asked to refrain from exercise for a 48‐h period prior to testing. Participants were instructed to lie supine on a therapeutic bench and remain still throughout the RMR test. Participants were tested in a private room with the lights dimmed. Average measurements were recorded every 30 s for a 30‐min span. The final 15 min of RER measurements were used to estimate substrate oxidation at rest.

Submaximal substrate oxidation was estimated using the RER from the TrueMax 2400 Metabolic Measurement Cart (ParvoMedics) during an acute bout of exercise. Participants entered the laboratory following a specified dietary protocol and refraining from exercise for a 48‐h period prior to the assessment. In short, participants ate a standardized breakfast 2 h before their visit which consisted of set volumes of cheerios, milk, and orange juice. The macronutrient breakdown of this meal consisted of 4.76 grams of fat (14.2% of total calories), 55.48 grams of carbohydrates (73.4% of total calories), and 9.37 grams of protein (12.4% of total calories). Participants recorded their food and beverage consumption at baseline to ensure standardization of post‐testing. Participants were instructed to consume the same food (recorded the day prior to baseline testing) prior to their post‐testing visit. The dietary protocol was performed to ensure participants’ substrate oxidation values were not altered based on their dietary consumption at baseline and post‐testing.

Participants were then asked to cycle at a cadence of 80–90 revolutions per minute for 4 to 6 min until steady state was reached at 50% of their VO_2_ and heart rate reserve. VO_2peak_ was assessed at baseline and post‐testing; thus, allowing us to alter workloads relative to the participants’ VO_2peak_ values during both testing periods. Steady‐state was defined as the following: heart rate = ±5 beats per minute (CSEP, 2013) and relative VO_2_ = ±1.2 ml/kg/min using 30 s averages over a minute period, based on previous literature (Myers et al., 1990) and pilot data obtained in the laboratory. Heart rate measurements were recorded using Zephyr^TM^ BioHarness and OmniSense system (Medtronic). The RER values during the final steady‐state minute of the test were averaged to estimate submaximal substrate oxidation.

### 2.7 Outcome measure—insulin sensitivity

An OGTT was used to estimate insulin sensitivity at baseline and post‐testing. The OGTT has been shown as appropriate for estimating insulin sensitivity in individuals without diabetes mellitus with reasonable accuracy (Stumvoll et al., 2000). The OGTT was performed following a 12‐h fast. Participants were instructed to consume a 75 g Trutol^TM^ Glucose Tolerance Test Beverage. Participants had 1‐min to consume the whole beverage. A 2‐h timer was started following the complete consumption of the beverage and blood was taken at the following timepoints: 0 (before drinking the 75g Trutol^TM^), 30, 60, and 120 min. Blood was collected in a vacutainer via intravenous blood draws, which were performed by a registered nurse. Blood samples were then centrifuged for 15 min at a spin rate of 2,000 g at 4℃ to separate the plasma from the blood sample. Plasma was then collected and stored at −80℃ until further analysis was performed.

Measurements of insulin and glucose for each timepoint were analyzed using an Insulin Enzyme‐Linked Immunoassay (ELISA) Kit (Catalog # 90095) and Glucose Assay Kit (Catalog # 81696) respectively from Crystal Chem (Illinois, USA) in accordance with the manufacturer's protocol. Intra‐assay coefficient of variation was 4.6% for glucose and 4.3% for insulin. Optical density of the plated samples was measured using an EPOCH 2 Microplate Spectrophometer (Biotek). Gen5 software was used to analyze the optical density of samples with the specified wavelength in accordance with the manufacturer's protocol. Insulin sensitivity was estimated using the Matsuda index with the derived insulin and glucose measurements from the OGTT (Matsuda & DeFronzo, 1999). The formula for the Matsuda index is as follows: Insulin Sensitivity Index = 10,000/√(fasting plasma glucose concentration*fasting plasma insulin concentration*mean plasma glucose concentration*mean plasma insulin concentration) (Gutch et al., 2015). Additionally, total insulin and total glucose area under the curve (AUC) was measured using the trapezium methods (Le Floch et al., 1990).

### 2.8 Exploratory variables

Anthropometric measurements, body composition, lipid profile, cardiorespiratory fitness, and habitual physical activity levels (measured via Piezo RX Pedometers) were measured for exploratory purposes and sample description. BMI and waist circumference were measured using the CSEP protocol (CSEP, 2013). Briefly, participants’ height and weight were measured to the nearest 0.5 cm and 0.1 kg respectively using a calibrated column scale (SECA^®^ model #213). Height was measured using a standardized stadiometer. Participants stood straight up with their feet together, arms to their side, and with no shoes while the measurement was taken, following an inhalation (CSEP, 2013). Participants’ weight was measured with minimal clothing (shorts and t‐shirt), while waist circumference measurement was taken after a normal expiration at the upper lateral border of the iliac crest (CSEP, 2013). Participants stood with their feet shoulder‐width apart and their arms crossed on their chest (CSEP, 2013). An anthropometric tape measure was used for measuring waist circumference recorded to the nearest 0.5 cm (CSEP, 2013).

Body composition was estimated using the BodPod version 1.69 (COSMED). Participants wore tight shorts and a bathing cap while sitting still and breathing normally during the BodPod test. Based on participants’ body density values, fat‐free mass and fat mass was estimated using the Siri formula [% Body Fat = (495 / Body Density) – 450]. The BodPod has been shown to be a valid and reliable tool to estimate body composition as body fat error scores typically range from 1% to 2.7% (Vescovi et al., 2001).

Lipid profile was measured using CardioChek PA Analyzer as validated by the National Cholesterol Education Program of the National Institutes of Health (CRMLN & NCEP,); reliability reported previously (Bastianelli et al., 2017; Gao et al., 2016). Research staff cleaned participants’ left ring finger using an alcohol swab. Two collection tubes were filled and transferred to the testing strip for analysis. Measurements included total cholesterol, high‐density lipoprotein cholesterol, triglyceride levels, low‐density lipoprotein cholesterol, and glucose levels.

Cardiorespiratory fitness was measured by performing a graded cycle ergometer exercise test using the TrueMax 2400 Metabolic Measurement Cart (ParvoMedics). The protocol was as follows: 5 min of cycling at 50 watts followed by increasing resistance by 25 watts each minute until exhaustion at 80–90 revolutions per minute. VO_2peak_ values were used to estimate cardiorespiratory fitness by averaging the final 30 s over 5‐s measurements; heart rate, RER, and rate of perceived exertion was also recorded throughout the test.

### 2.9 Statistical analysis

A power calculation for a mixed‐model ANOVA was performed to determine the appropriate sample size. The sample size was calculated using G‐power software (Version 3.1.9.2, Germany) with a large effect size of 0.4, an alpha of 0.05, and a power of 0.8. The total sample size per group was eight. Studies reported that 50% of individuals dropout within the first six months of exercise training (Linke et al., 2011). Given reporting that high intensity interval training (HIIT) interventions have a much lower (18%) dropout rate (Reljic et al., 2019) and the duration of the current intervention was only four weeks, we aimed to recruit a minimum of eight additional participants per group. To account for dropout rate, to ensure that we could adjust for confounding variables, and considering our recruitment rate, our final sample size was approximately 17 per group (total 34).

Shapiro–Wilk tests were performed to test for normality within the sample and a visual exploration of the data was performed which confirmed normality. General characteristics are presented as mean ± SD for continuous variable and N (%) for categorical variables. Differences in baseline and post‐testing values stratified by BMI groups were analyzed using paired sample *t*‐tests. Then, a mixed model ANOVA was performed to see whether there was a significant interaction effect between time and BMI classification with changes in insulin sensitivity (Matsuda index) and changes in substrate oxidation at rest and during submaximal exercise. The mixed‐model ANOVA for substrate oxidation was adjusted for cardiorespiratory fitness, which has been suggested to impact substrate oxidation (Gaitán et al., 2019). In contrast, adjustment for fat‐free mass was performed in the mixed‐model ANOVA for insulin sensitivity (DeFronzo et al., 1985). Bivariate Pearson's correlations were performed to investigate if changes in substrate oxidation at rest and during submaximal exercise were correlated with changes in insulin sensitivity. Data management and statistical analyses were performed using SPSS version 26 and STATA 16.1 software (StataCorp). A *p* ≤ 0.05 was considered significant.

## 3 RESULTS

Table 1 outlines the descriptive characteristics of the sample stratified by BMI groups with the exception of age and sex. Briefly, the average age of individuals without obesity was 41.7 ± 13.3 years, while individuals living with obesity was 39.3 ± 11.8 years (*p* > 0.05). The proportion of males was 50% and 37.5% (*p* > 0.05) for those without obesity and living with obesity, respectively. Significant reductions in body fat percentage and resting systolic blood pressure were observed in individuals living with obesity, while a significant increase in cardiorespiratory fitness was observed in individuals without obesity (all *p* < 0.05). No significant difference was observed in lipid profile between and within groups (*p* > 0.05), while several measurements of insulin and glucose measurement from the OGTT were significantly different within both groups (*p* < 0.05). AUC analysis for insulin significantly increased for those without obesity (*p* < 0.05); however, no difference was observed for individuals living with obesity (*p* > 0.05). For glucose, AUC analysis revealed no change following SIT for those without obesity (*p* > 0.05); however, individuals living with obesity reduced their AUC value for glucose (*p* < 0.05). No difference in physical activity levels (*p* < 0.05) and RMR (*p* < 0.05) were observed within and between groups. No significant change in submaximal VO_2peak_ was observed following SIT in either group (*p* > 0.05). No significant difference in VO_2_ (*p* > 0.05) and VCO_2_ (*p* > 0.05) measures during the test of substrate oxidation at rest and during submaximal exercise were observed within and between groups. Finally, no significant difference was observed in substrate oxidation rates in individuals without obesity (*p* > 0.05). However, significant increases in submaximal fat oxidation rate were observed for individuals living with obesity, while reductions in carbohydrate oxidation at rest and during submaximal exercise were observed in this group as well (all *p* < 0.05).

**TABLE 1:** General characteristics of individuals living without and with obesity

	Without Obesity (*n* = 18)			Living with Obesity (*n* = 16)		
	Pre	Post	*p*	Pre	Post	*p*
Anthropometrics						
Weight (kg)	68.3 ± 10.5	68.7 ± 10.0	0.051	104.0 ± 18.7	103.7 ± 18.8	0.638
Body mass index (kg/m^2^)	22.9 ± 1.6	23.0 ± 1.5	0.116	34.1 ± 3.8	34.1 ± 4.0	0.737
Waist circumference (cm)	85.0 ± 7.1	84.9 ± 7.7	0.872	113.2 ± 12.4	112.2 ± 13.0	0.165
Body fat (%)	24.7 ± 9.5	24.3 ± 9.5	0.221	42.6 ± 6.5	41.7 ± 7.2	0.048
Fat mass (kg)	16.7 ± 6.7	16.4 ± 6.6	0.254	44.4 ± 11.8	43.5 ± 12.4	0.109
Fat‐free mass (kg)	51.4 ± 10.9	51.9 ± 10.9	0.067	59.3 ± 10.7	60.6 ± 12.2	0.076
Metabolic Profile						
Resting SBP (mmHg)	107.4 ± 7.1	104.4 ± 10.7	0.216	124.9 ± 14.2	116.5 ± 13.1	0.003
Resting DBP (mmHg)	68.0 ± 9.3	68.0 ± 6.4	0.987	83.8 ± 7.5	80.3 ± 6.9	0.084
Total Chol (mmol/L)	4.8 ± 1.6	4.7 ± 1.0	0.501	5.5 ± 1.4	5.2 ± 0.9	0.164
HDL Chol (mmol/L)	1.6 ± 0.4	1.6 ± 0.4	0.767	1.4 ± 0.3	1.3 ± 0.3	0.541
Triglycerides (mmol/L)	1.2 ± 0.8	1.0 ± 0.4	0.103	1.7 ± 0.9	1.5 ± 0.7	0.156
LDL Chol (mmol/L)	2.8 ± 1.2	2.6 ± 0.9	0.472	3.4 ± 1.3	3.2 ± 0.8	0.341
Fasting glucose (mmol/L)	4.8 ± 0.6	4.9 ± 0.5	0.741	5.2 ± 0.7	5.2 ± 0.8	0.908
OGTT Results						
Insulin T0 (pmol/L)	12.0 ± 11.6	24.2 ± 17.4	0.005	52.5 ± 29.3	65.7 ± 35.1	0.068
Insulin T30 (pmol/L)	144.7 ± 65.2	242.0 ± 167.3	0.019	442.5 ± 169.4	444.3 ± 320.7	0.976
Insulin T60 (pmol/L)	207.3 ± 74.3	265.2 ± 172.6	0.214	618.1 ± 217.3	476.8 ± 357.8	0.048
Insulin T120 (pmol/L)	126.1 ± 74.3	164.5 ± 72.7	0.025	413.5 ± 207.4	391.8 ± 272.8	0.733
Glucose T0 (mmol/L)	4.5 ± 1.0	4.5 ± 0.8	0.926	5.0 ± 1.0	4.8 ± 0.7	0.474
Glucose T30 (mmol/L)	6.2 ± 1.5	5.7 ± 1.2	0.243	7.2 ± 1.4	5.8 ± 1.2	0.013
Glucose T60 (mmol/L)	5.2 ± 1.6	5.2 ± 1.7	0.557	7.6 ± 2.5	6.0 ± 1.7	0.016
Glucose T120 (mmol/L)	4.4 ± 1.5	4.2 ± 1.2	0.457	5.5 ± 1.6	5.5 ± 1.6	0.856
AUC insulin	17634 ± 6437.4	24523 ± 13335	0.032	54278 ± 18807	48506 ± 31272	0.336
AUC glucose	621.9 ± 132.6	595.4 ± 118.9	0.408	800.7 ± 190.4	678.6 ± 145.4	0.029
Activity Level and Fitness						
MVPA (min/week)	127.2 ± 121.0	96.5 ± 143.0	0.330	14.0 ± 20.0	15.8 ± 21.9	0.987
CRF (ml/kg/min)	36.4 ± 8.6	39.1 ± 8.7	0.004	25.8 ± 5.4	26.6 ± 4.8	0.352
RMR (kcal/day)	1539.2 ± 294.9	1568.6 ± 255.0	0.558	2062.2 ± 327.7	2100.1 ± 272.7	0.337
SS VO_2peak_ (ml/kg/min)	19.9 ± 4.8	21.0 ± 4.3	0.090	14.8 ± 2.35	15.1 ± 2.7	0.422
Substrate Oxidation Rate						
Resting FO (g/min)	76.6 ± 25.9	73.1 ± 20.6	0.666	100.6 ± 27.7	105.7 ± 37.9	0.642
Submaximal FO (g/min)	168.0 ± 137.4	154.0 ± 167.0	0.624	105.5 ± 183.9	208.0 ± 130.1	0.009
Resting CHO (g/min)	94.4 ± 54.5	101.8 ± 53.3	0.675	132.7 ± 74.0	101.1 ± 53.8	0.032
Submaximal CHO (g/min)	1392.4 ± 534.6	1536.4 ± 434.5	0.205	1726.0 ± 748.5	1440.0 ± 749.5	0.024

Note

Continuous variables are presented as means ±standard deviation. Categorical variables are presented as n (%). Alpha level at 0.05.

Abbreviations: AUC, area under the curve; CHO, carbohydrate oxidation; Chol, cholesterol; CRF, cardiorespiratory fitness; DBP, diastolic blood pressure; FO, fat oxidation; HDL, high‐density lipoprotein; LDL, low‐density lipoprotein; MVPA, moderate‐to‐vigorous physical activity; OGTT, oral glucose tolerance test; RMR, resting metabolic rate; SBP, systolic blood pressure; SS, steady state.

**FIGURE 3:**
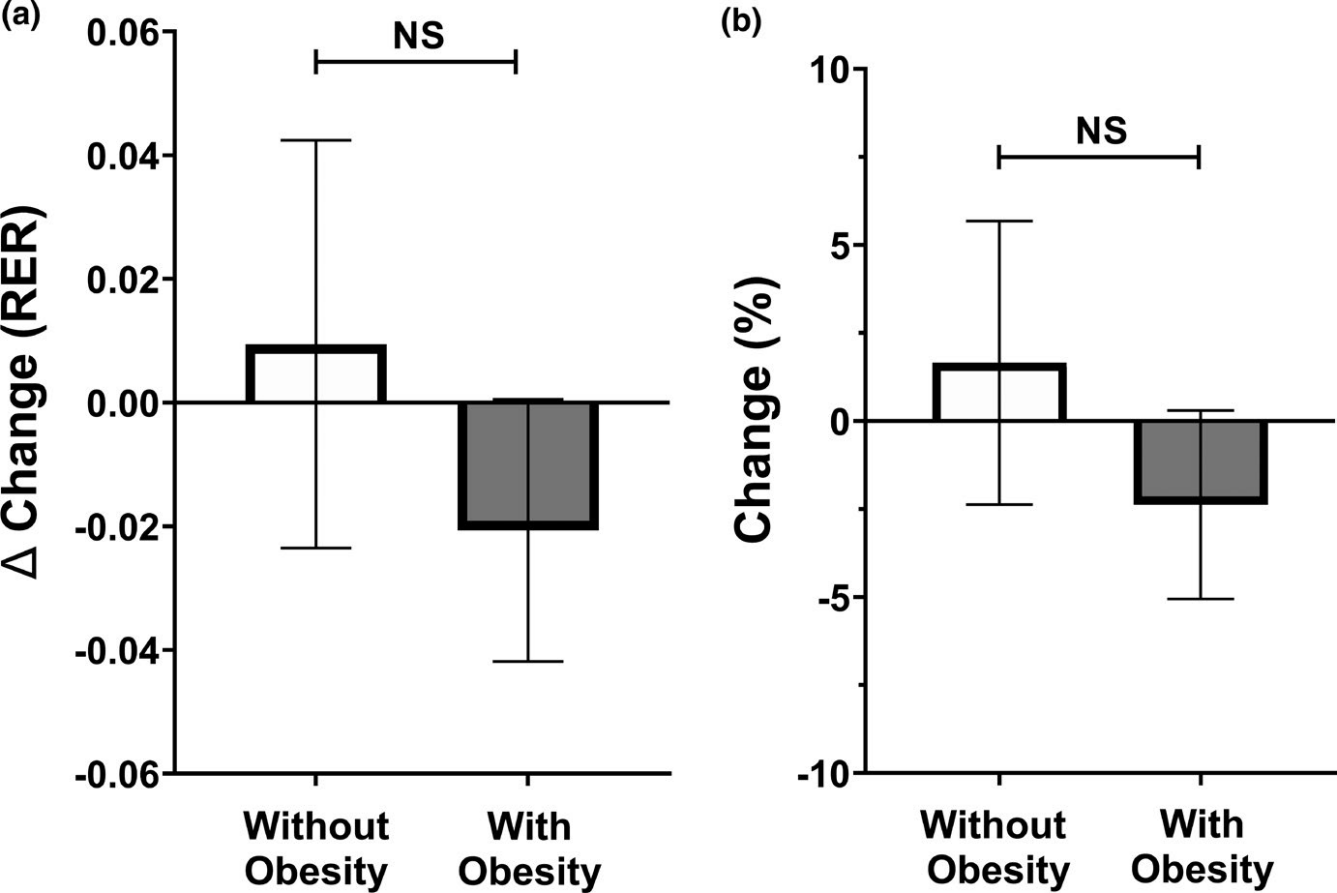
(a, b) Absolute change and percentage change in substrate oxidation at rest. Data are presented as mean and 95% confidence intervals. Significant difference was considered *p* ≤ 0.05

**FIGURE 4:**
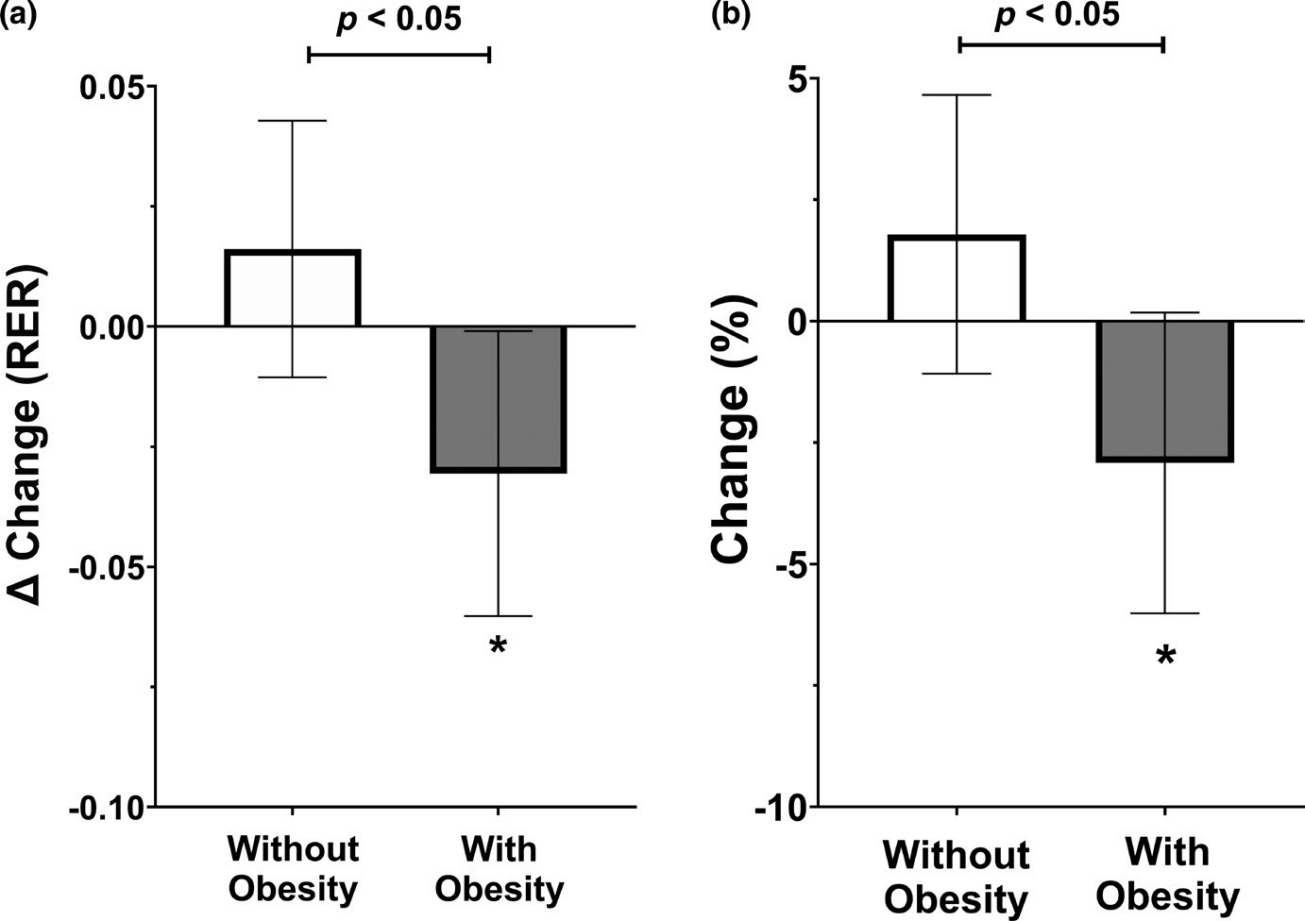
(a, b) Absolute change and percentage change in submaximal substrate oxidation. Data are presented as mean and 95% confidence intervals. * represents significant difference from baseline (*p* ≤ 0.05)

**FIGURE 5:**
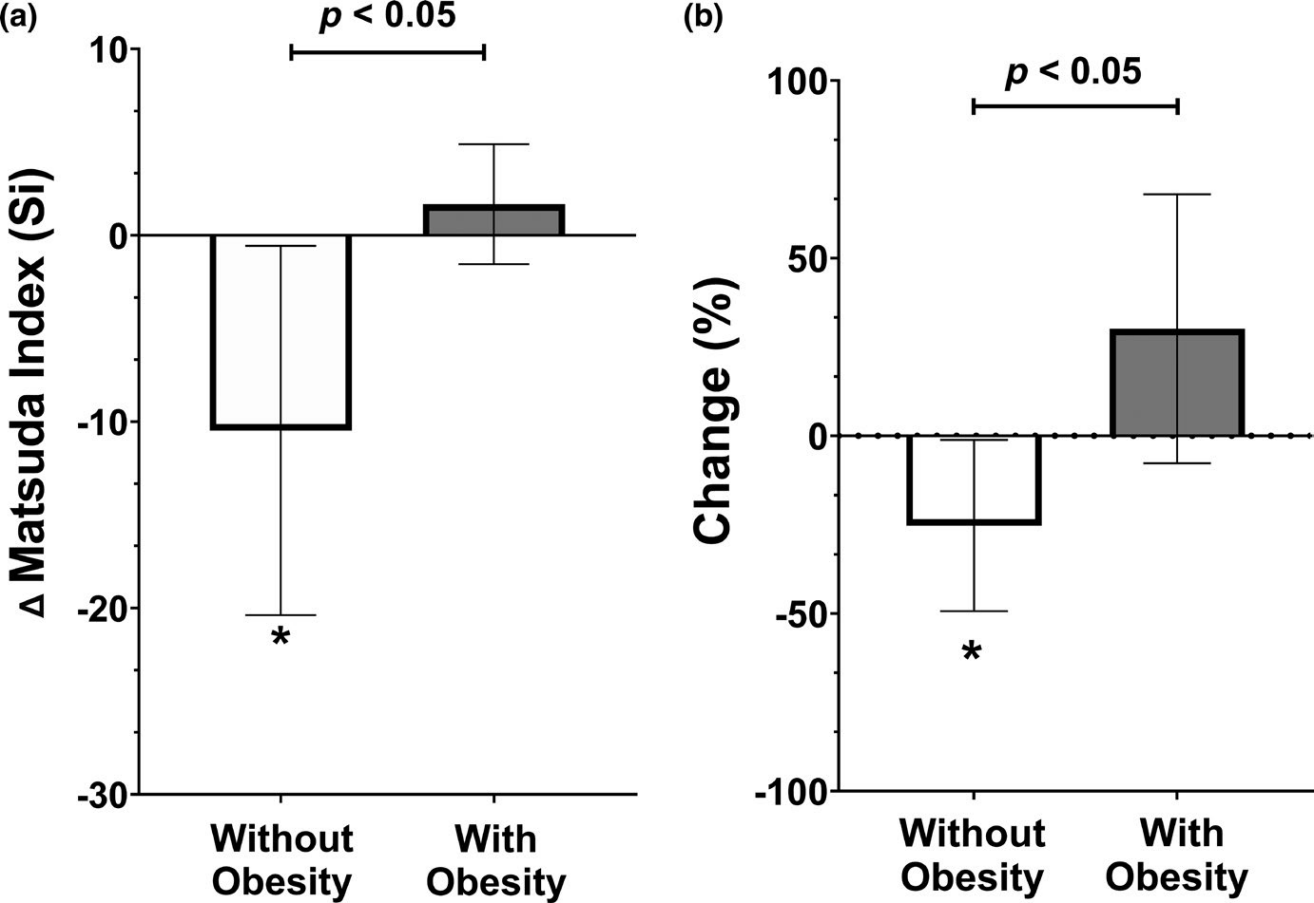
(a, b) Absolute change and percentage change in insulin sensitivity (Si) from the Matsuda Index. Data are presented as mean and 95% confidence intervals. * represents significant difference from baseline (*p* ≤ 0.05)

Figure 3a,b and Figure 4a,b present the impact of SIT on substrate oxidation at rest and during submaximal exercise, defined as changes in RER. No significant changes or differences in substrate oxidation at rest were observed between individuals without obesity (baseline: 0.80 ± 0.05, post: 0.81 ± 0.04) and those living with obesity (baseline: 0.80 ± 0.04, post: 0.78 ± 0.04) (*p* > 0.05). However, submaximal substrate oxidation improved significantly (reduction in RER) following four weeks of SIT in individuals living with obesity (baseline = 0.95 ± 0.08, post = 0.92 ± 0.06; *p* < 0.05), a change that was significantly different (*p* < 0.05) from individuals without obesity (baseline = 0.93 ± 0.04, post: 0.94 ± 0.05). Figure 5a,b describe the absolute and percent changes in insulin sensitivity (estimated via Matsuda index) following four weeks of SIT. Individuals without obesity had a significant reduction in insulin sensitivity by 25.3% (baseline = 29.2 ± 18.4, post: 18.7 ± 14.2; *p* < 0.05) after only four weeks of SIT, while individuals living with obesity had an increase of 30.1% in insulin sensitivity, although it did not reach statistical significance (baseline = 5.9 ± 3.4, post: 7.5 ± 7.0; *p* > 0.05). Significant differences were observed for absolute and percent changes in insulin sensitivity between groups (*p* < 0.05).

When adjusted for cardiorespiratory fitness, no significant BMI group effect [F(1,30) = 0.564, *p* = 0.458], time effect [F(1,30) = 0.362, *p* = 0.552], or interaction effect [F(1,30) = 0.851, *p* = 0.364] was observed for substrate oxidation at rest. There was no significant BMI group effect [F(1,31) = 0.087, *p* = 0.773] observed for submaximal substrate oxidation when adjusted for cardiorespiratory fitness; however, there was a significant time effect [F(1,31) = 6.340, *p* = 0.017] and interaction effect [F(1,31) = 14.55, *p* = 0.001] observed within the model. Finally, there was no significant time effect [F(1,31) = 0.008, *p* = 0.927] observed for insulin sensitivity when adjusted for fat‐free mass; however, there was a significant BMI group effect [F(1,31) = 27.740, *p* < 0.001] and interaction effect [F(1,31) = 5.010, *p* = 0.033] observed. Additionally, we adjusted each model for fat mass, VO_2peak_, age, and sex which yielded similar results for all analyses.

**FIGURE 6:**
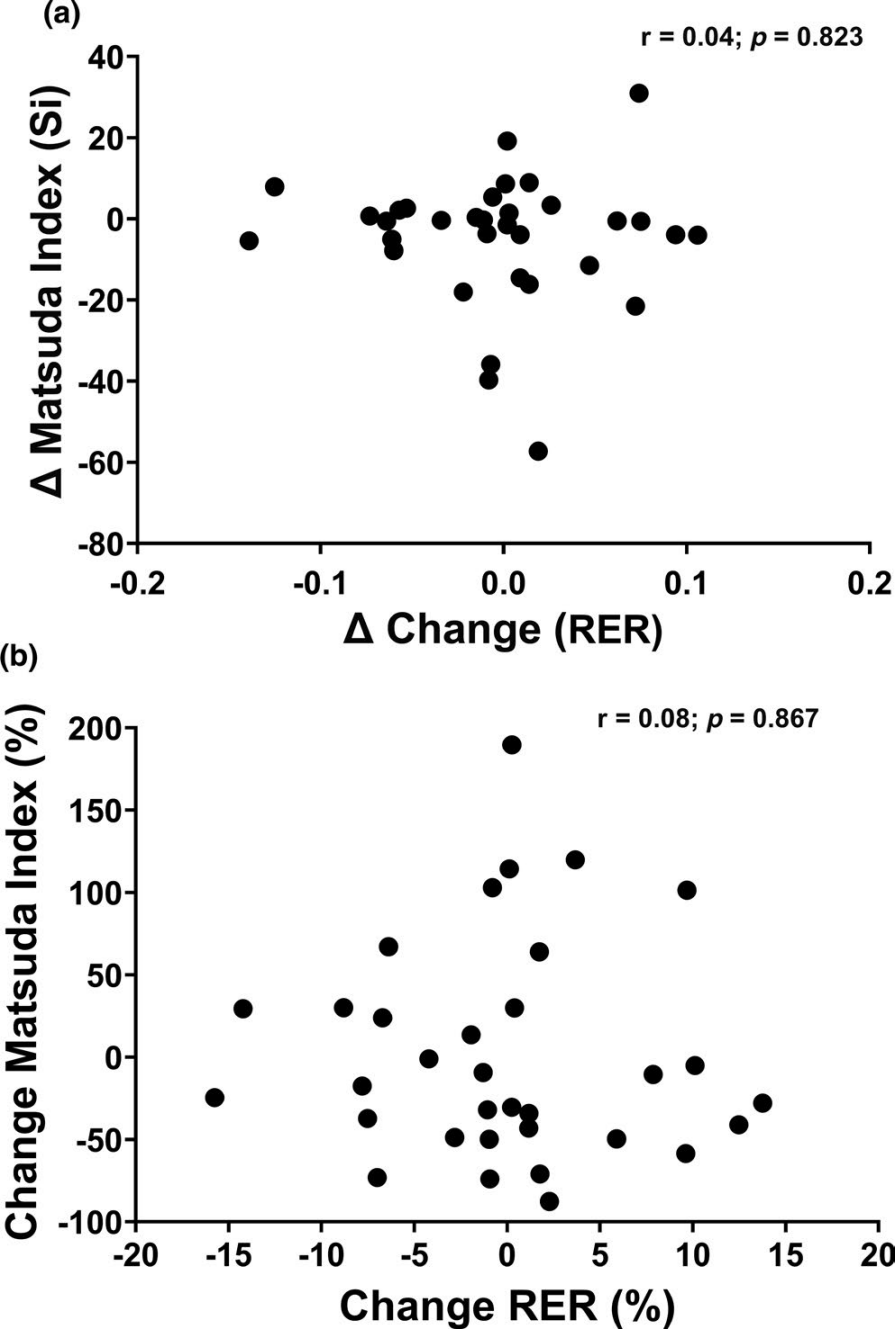
(a, b) Association between absolute changes and percentage changes in substrate oxidation at rest and insulin sensitivity for the whole sample

**FIGURE 7:**
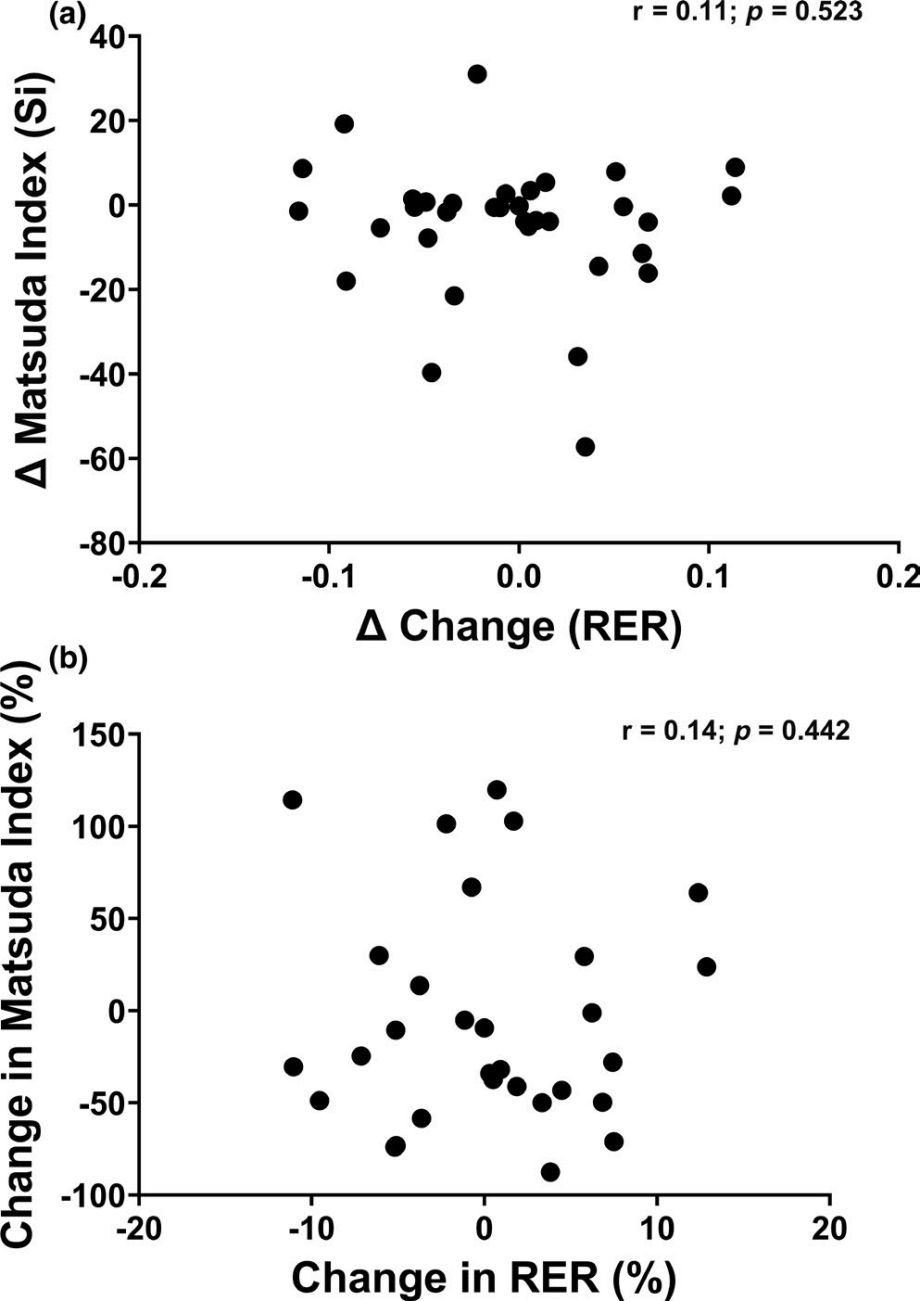
(a, b) Association between absolute changes and percentage changes in submaximal substrate oxidation and insulin sensitivity for the whole sample

Figure 6a,b and Figure 7a,b examine the correlations between absolute and percent changes in substrate oxidation at rest and during submaximal exercise, as well as absolute and percent changes in insulin sensitivity. Overall, no significant correlations were observed between changes in substrate oxidation at rest and changes in insulin sensitivity (Figure 6a: *p* > 0.05) or between percent change in substrate oxidation at rest and percent change in insulin sensitivity (Figure 6b: *p* > 0.05). Similar results were observed for absolute and percent changes in submaximal substrate oxidation and insulin sensitivity (Figure 7a,b: *p* > 0.05). Correlations were also stratified by BMI group; however, no significance was observed (*p* > 0.05).

## 4 DISCUSSION

The main objectives of this study were (1) to investigate changes in insulin sensitivity and substrate oxidation at rest and during submaximal exercise following a four‐week SIT intervention in individuals living with or without obesity, and (2) to investigate whether changes in substrate oxidation following a four‐week SIT intervention were associated with changes in insulin sensitivity. The main finding of our study suggests that individuals living with obesity increased fat oxidation during submaximal exercise compared to individuals without obesity following only four weeks of SIT. Yet, no changes in substrate oxidation at rest were observed in either group. Although a significant interaction effect was observed for insulin sensitivity, this was likely due to a reduction in insulin sensitivity for individuals without obesity, and not from an improvement in individuals living with obesity. No correlations were observed between changes in substrate oxidation at rest and during submaximal exercise, and changes in insulin sensitivity.

These findings provide important insight into the impact of short‐term SIT on substrate oxidation and insulin sensitivity in individuals living with and without obesity; an important comparison given the numerous cardiometabolic differences between the two BMI groups. Here we show that an intervention of short duration, as low as four weeks, is sufficient for altering substrate oxidation in individuals living with obesity to a greater extent than in individuals without obesity, suggesting SIT increases sensitivity to metabolic shifts in individuals living with obesity. Although fat oxidation during submaximal exercise is enhanced, this does not lead to adaptation at rest in individuals living with obesity. Alterations in substrate oxidation do not appear to be the mechanism by which four weeks of SIT improves insulin sensitivity in individuals living with obesity. Although arbitrary, the use of four weeks is not trivial, as previous literature has suggested that substrate oxidation at rest and during submaximal exercise change following four and six weeks of SIT respectively (Alkahtani et al., 2013; Jabbour & Iancu, 2017). Together, these data provide relevant information to enhance the design of training strategies targeting obesity and obesity‐related cardiometabolic management.

A significant increase in submaximal fat oxidation in individuals living with obesity was observed compared to individuals without obesity following SIT. This finding aligns with previous research that observed increases in submaximal fat oxidation following exercise training (Aggel‐Leijssen et al., 2001, 2002; Dumortier et al., 2003; Jabbour & Iancu, 2017; Kanaley et al., 2001). These studies have mainly focused on the impact of continuous aerobic exercise on submaximal substrate oxidation (Gutch et al., 2015; Kanaley et al., 2001; Le Floch et al., 1990; Vescovi et al., 2001). Similar adaptations in substrate oxidation with lower volumes of interval training compared to continuous endurance exercise are reported (Alkahtani et al., 2013; Astorino et al., 2013; Burgomaster et al., 2008). In a randomized controlled trial by Jabbour & Iancu (2017), an increase in submaximal fat oxidation in individuals living with obesity during exercise at multiple absolute workloads (25, 50, and 75 Watts) following six weeks of SIT was observed (Jabbour & Iancu, 2017). However, using absolute workloads rather than relative workload (i.e. % VO_2max_) is a potential limitation as SIT and HIIT are effective at improving cardiorespiratory fitness when compared to continuous exercise (Daussin et al., 2008; Gist et al., 2014; Sloth et al., 2013; Upadhyay et al., 2010). Thus, participants likely worked at a reduced relative intensity during post‐testing in comparison to baseline testing. In turn, the alteration in submaximal substrate oxidation may solely be a result of a reduction in intensity and not of SIT itself. It is important to note that no change in cardiorespiratory fitness level was observed in the study for the whole cohort (Jabbour & Iancu, 2017), which may mitigate this concern. However, based on changes in cardiorespiratory fitness, 15 participants from the present study worked at different workloads at post‐testing compared to baseline testing. Therefore, our study adds to the current literature by providing more accurate information using a stronger design, while observing an increase in submaximal fat oxidation following SIT.

A significant change in insulin sensitivity in individuals living with obesity following SIT was observed compared to individuals living without obesity; however, this finding was likely due to a 25% reduction in insulin sensitivity for individuals without obesity. Our findings disagree with previous literature that suggests improvements in insulin sensitivity following exercise training. (Jabbour & Iancu, 2017; Jelleyman et al., 2015; Malin et al., 2013; Matos et al., 2018; Richards et al., 2010; Sandvei et al., 2012; Whyte et al., 2010). In a meta‐analysis performed by Jelleyman et al. (2015), the authors identified a significant reduction in insulin resistance following HIIT (standardized mean difference of −0.33 95%CI [−0.47 ‐ −0.18]), although these data are not exclusively from studies investigating samples of participants living with obesity and SIT (Jelleyman et al., 2015). Additionally, individuals without obesity had an increase in insulin AUC, while glucose AUC did not significantly change from baseline. These data could suggest that participants without obesity released more insulin to maintain blood sugar levels, reinforcing the deterioration in glucose metabolism. Furthermore, other exercise trials suggested that some individuals might have a negative cardiometabolic and insulin sensitivity response to exercise training (Blizzard LeBlanc et al., 2017; Bouchard et al., 1999; Matos et al., 2018; Sénéchal et al., 2015). It has been suggested that eight weeks of HIIT does not elicit a reduction of insulin resistance for individuals living with obesity without pre‐existing insulin resistance, whereas significant improvements are observed in individuals living with obesity and insulin resistance (Matos et al., 2018). Altogether, these findings suggest that different forms of HIIT and SIT are impactful at altering insulin sensitivity in populations with increased insulin resistance; however, they may not be as beneficial for individuals without pre‐existing insulin resistance. Interestingly, although participants living with obesity were normoglycemic, a significant decrease in the AUC for glucose was observed whereas no change was observed in the AUC for insulin. This suggests that for a given amount of insulin, more glucose was taken up by the peripheral tissue. Even though no significant improvement in insulin sensitivity was observed for those living with obesity, these data shed light on potential improvements in glucose metabolism in this BMI group.

No change was observed in substrate oxidation at rest following four weeks of SIT in either group. This observation is surprising as previous research observed alterations in substrate oxidation at rest following continuous exercise training in individuals living with obesity (Goodpaster et al., 2003; Malin et al., 2013; Potteiger et al., 2008). Nevertheless, individuals living with obesity in the current study did not present a significant difference in baseline fat oxidation at rest compared to individuals without obesity (0.80 ± 0.04 compared to 0.80 ± 0.05; *p* > 0.05), whereas Kelley et al. (1999) previously reported a larger difference between these groups (0.90 ± 0.01 compared to 0.83 ± 0.02; *p* < 0.01) (Kelley et al., 1999). Therefore, it can be speculated that participants living with obesity in our study did not have abnormal substrate oxidation at baseline, which may explain the lack of change observed for this outcome. Furthermore, Whyte et al. (2010) concluded that there was a significant reduction in RQ (0.78 ± 0.01 to 0.73 ± 0.01; *p* < 0.05) following SIT, suggesting an increase in fat oxidation at rest (Whyte et al., 2010). However, Whyte et al. (2010)’s findings did not incorporate a comparison group or females, such that it may be too confounded or under‐powered to be certain of the impact on substrate oxidation (Lundsgaard & Kiens, 2014; Whyte et al., 2010).

No significant correlations were observed between changes in insulin sensitivity and changes in substrate oxidation at rest and during submaximal exercise, which is inconsistent with previous literature. Originally, Kelley et al., (1999) reported a relationship between insulin sensitivity and substrate oxidation following 14 kg of weight loss (Kelley et al., 1999). This association between substrate oxidation and insulin sensitivity was also observed by Malin et al. (2013) following 12 weeks of high intensity exercise training‐induced weight loss in individuals living with obesity and insulin resistance (Malin et al., 2013). In our study, no significant weight loss was observed following SIT and participants were normoglycemic with a healthy metabolic profile. Therefore, discrepancies between our results and previous data might be explained by the lack of exercise induced‐weight loss and an abnormal metabolic profile. Altogether, these findings suggest that exercise‐induced weight‐loss is important for the association between improvements in substrate oxidation and insulin sensitivity compared to exercise training itself.

### 4.1 Strengths and limitations

Although the present study provides important insight for the management of obesity and obesity‐related cardiometabolic risk factors, some limitations require acknowledgment. First, no non‐experimental control group was used; thus, limiting the conclusions that could be drawn from the study. Furthermore, having a small sample size may have impacted the level of significance observed in the main statistical analysis and limited the number of confounders that were adjusted for in the models. Participants may have only reached a VO_2peak_ rather than a true VO_2max_ measurement during the cardiorespiratory test, which may have impacted the relative exercise intensity for the submaximal substrate oxidation measurement. Also, the use of MVPA as an exclusion criterion for only one group may have impacted the findings. However, the analysis for substrate oxidation was adjusted for cardiorespiratory fitness, which we believe is increasingly important compared to MVPA levels. Given the differing body composition, using 7.5% of total body weight may have resulted in a greater stimulus for individuals living with obesity in the current study. However, it is important to note that no difference was observed between groups in total work volume (measured in kgm; *p* > 0.05) throughout the SIT intervention; thus, mitigating this limitation. Additionally, previous training experience was not recorded for either group, which may impact the findings observed. Finally, baseline substrate oxidation at rest was similar between groups which may have impacted the findings regarding substrate oxidation at rest. Nevertheless, the study is strengthened by its design allowing for the comparison of individuals without obesity and individuals living with obesity, which has not been performed prior to the current trial. Furthermore, an objective quantification of insulin sensitivity was used to ensure exclusion of individuals with impaired glucose tolerance. Finally, SIT was supervised in a one‐on‐one environment by the same researchers over the four‐week period, allowing the study to have a tightly controlled environment for the exposure variable.

## 5 CONCLUSION

In summary, four weeks of SIT elicited significant increases in submaximal fat oxidation in individuals living with obesity compared to individuals without obesity. No significant change was observed in substrate oxidation at rest and insulin sensitivity for individuals living with obesity. Changes in substrate oxidation were not correlated with changes in insulin sensitivity. Future research should focus on mechanistic studies to better understand how changes in substrate oxidation and insulin sensitivity are modified using SIT.

## CONFLICT OF INTEREST

Conflict of interest: The authors declare there are no conflict of interest.

## AUTHOR CONTRIBUTIONS

BHC contributed to conceiving the main idea and design of the study, data collection and analysis, and drafting and editing the manuscript. KS contributed to conceiving the main idea and design of the study, data analysis, and drafting and editing the manuscript. ALE and KRB contributed to data analysis and editing the manuscript. DRB contributed to drafting and editing the manuscript. MS contributed to conceiving the main idea and design of the study, data analysis, and drafting and editing the manuscript.
